# A two-sample mendelian randomization analysis investigates associations between gut microbiota and infertility

**DOI:** 10.1038/s41598-023-38624-6

**Published:** 2023-07-15

**Authors:** Taozhi Li, Wenbo Shao, Yukun Wang, Rui Zhou, Zhangjun Yun, Yalin He, Yu Wu

**Affiliations:** 1 Department of Oncology, Xiyuan Hospital, China Academy of Chinese Medical Sciences, Beijing, China; 2 Department of Cardiology, Guang’anmen Hospital, China Academy of Chinese Medical Sciences, Beijing, China; 3 Beijing University of Chinese Medicine, Beijing, China; 4 Chongqing Jiangjin District Hospital of Traditional Chinese Medicine, Chongqing, China

**Keywords:** Microbiology, Health care

## Abstract

Observational studies have provided evidence of a correlation between alterations in gut microbiota composition and infertility. However, concrete proof supporting the causal relationship is still lacking. We performed a Mendelian randomization study to assess whether genetically gut microbiota composition influences the risk of infertility. The genetic data pertaining to gut microbiota were obtained from a genome-wide association study meta-analysis, which was conducted among 24 cohorts (18,340 participants) from the international MiBioGen consortium. By the primary method of assessing causality, we have identified 2 family taxa, 2 genus taxa, and 1 order taxa that were linked to a low risk of male infertility, while 1 genus taxa were associated with a high risk of male infertility. Furthermore, we have discovered 6 genus taxa, 1 phylum taxa, 1 class taxa, 1 order taxa, and 1 family taxa that were associated with a low risk of female infertility, while 1 genus taxa were linked to a high risk of female infertility. This study successfully confirmed that there was a causal link between gut microbiota and infertility. The identification of these specific strains through genetic prediction offers a valuable insight for early diagnosis, prevention, and treatment of infertility.

## Introduction

China is currently experiencing a rapid aging population, coupled with a substantial rise in the prevalence of infertility among couples. According to infertility studies conducted in 2012, the percentage of childbearing couples facing infertility has increased significantly from 3% in 2009 to 12.5–15%, which was comparable to rates observed in industrialized nations ranging from 15 to 20%. Until now, the global impact of infertility is substantial, affecting approximately 186 million individuals worldwide^1^, and it has sparked numerous extensive discussions. Not only does infertility have a profound impact on patients and their families, but it also places a considerable financial burden on society. It is crucial for us to approach this issue with rationality and place greater emphasis on early identification and prevention of infertility. Any government that overlooks this matter will face severe consequences. Therefore, early identification and prevention strategies are of paramount importance in this context.

Nowadays, in the field of systems biology, the growing significance of gut microbiota has opened up new perspectives on the exploration of disease mechanisms. Thanks to advancements in high-throughput technologies^2^, we are now capable of analyzing hundreds of gut microbiota. The disruption of gut microbiota balance has been linked to a range of diseases^3^. Consequently, the study of gut microbiota has emerged as a crucial bridge in disease research, leading to continuous advancements in flora research technologies, from organelles to populations. As a result, exploring the relationship between gut microbiota and human diseases, including infertility, has sparked a great deal of interest^4^. Nevertheless, it is of great importance to note that there is still a lack of comprehensive analysis and in-depth understanding of the pathophysiological mechanisms underlying the flora, impeding a clear determination of the causal relationships of gut microbiota on infertility. Despite some observational epidemiological studies that have indicated a potential relationship between gut microbiota and male infertility^5, 6^, confirming a causal link through such studies remains challenging, this is primarily due to the presence of confounding factors and the possibility of reverse causality. The application of Mendelian randomization (MR) provides an effective approach to mitigate such biases by employing genetic variants that serve as proxies for exposure, enabling the exploration of causal relationships between risk factors and diseases^5^.

In particular, this approach effectively addresses the issue of reverse causality bias caused by unmodifiable genotype^5^. The underlying assumption is that genetic variants linked to the exposure of interest undergo random assortment during conception. By using genetic variants that serve as instrumental variables (IVs) to adjust for the influence of gut microbiota, we can assess the impact of gut microbiota on the incidence of infertility. Given the limited understanding of the causal relationship between gut microbiota and infertility, there is a significant knowledge gap in this area of research. In this study, we employed a two-sample MR framework to extensively investigate the causal effects of gut microbiota on infertility using summary data from genome-wide association study (GWAS). The primary objective of this research is to elucidate the underlying etiology of infertility related to gut microbiota and shed light on its associated biological mechanisms.

## Materials and methods

### Study design

**Figure 1:**
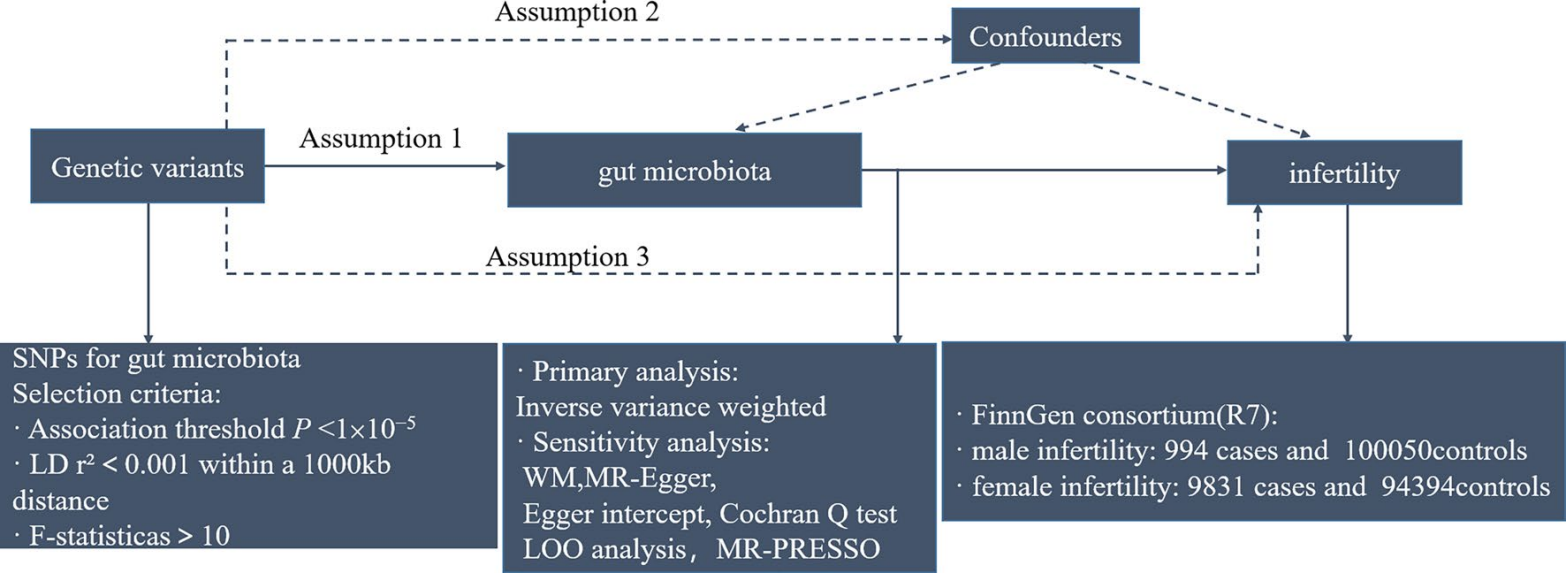
The workflow of Mendelian randomization study that exhibits causality between gut microbiota and infertility. Assumption 1, there is a significant association between genetic variation and exposure; Assumption 2, there is no correlation between genetic variation and confounding factors; Assumption 3, genetic variants exert effects on the outcomes by influencing the exposure of interest. SNPs, single nucleotide polymorphisms; LD, linkage disequilibrium; WM, weighted median; LOO, leave-one-out.

We provided convincing evidence for the causal effect between gut microbiota and infertility, based on GWAS summary statistics, using a two-sample MR design. This instrumental variable analysis shared similarities with randomized controlled trials (RCTs) in the random assignment of single nucleotide polymorphisms (SNPs) in offspring, without incorporating confounders such as age and gender. Furthermore, a rigorous MR study should comply with three assumptions: (1) IVs are strongly associated with the exposures of interest; (2) Genetic instruments are not associated with potential confounding factors (no statistical inference); and (3) there is interdependency between genetic instruments and the outcomes, accounting for exposure and confounders (The impact of genetic instruments on the outcomes is solely through the risk factors)^6^. An overview of the study was illustrated in Fig. 1.

### Exposure to GWAS‑gut microbiota

The genetic data pertaining to gut microbiota were obtained from a comprehensive case–control GWAS meta-analysis conducted by the international MiBioGen consortium^7^. This analysis involved the host genotypes and 16S fecal microbiome rRNA gene sequencing profiles assessed from a total of 24 cohorts (18,340 participants). The relevant cohorts were carried out in various countries, including the USA, Canada, Israel, South Korea, Germany, Denmark, the Netherlands, Belgium, Sweden, Finland, and the UK, respectively. Among these, twenty cohorts consisted of individuals from a single ancestry, with the majority being of European descent (16 cohorts, N = 13,266). The mean age range of the participants across the 24 cohorts ranged from 50 to 62 years (17 cohorts, N = 13,804). To investigate the impact of host genetics on the diversity of gut bacterial taxa, only the taxa met the criteria that presenting in more than 10% of the samples were included in each cohort. 15 microbial taxa without specific species names (family or genus unknown) were excluded, resulting in a total of 196 bacterial taxa being included in this study. The specific names of these taxa are provided in detail. The cohort dataset used in the study was adjusted for sex and age by the original investigators of the respective cohorts. All summary-level data utilized in the primary analysis were acquired from the publicly available at http://www.mibiogen.org.

### GWAS summary data for infertility

According to the classification of infertility by the World Health Organization(WHO), couples are diagnosed infertile if they fail to achieve pregnancy after one year of regular sexual activity^8^. Male factors contribute to as many as 50% of infertility cases in couples^9^. Therefore, GWAS summary data relevant to infertility were downloaded from the R7 release of the FinnGen consortium. The dataset included a total sample size of 994 cases and 100,050 controls for male infertility, and 9831 cases and 94,394 controls for female infertility, after excluding individuals with indeterminate sex, high genotype deficiency (> 5%), excess heterozygosity (± 4 standard deviation (SD)), and non-Finnish ancestry, respectively. Infertility diagnosis was based on the International Classification of Diseases codes N14 (8th, 9th, and 10th revisions). Detailed information on the GWAS studies employed in this research is provided in Table 1.

**Table 1:** Details of the GWASs included in the Mendelian randomization.

Consortium	Phenotype	Cases	Source
MiBioGen consortium	Gut microbiota	18,340	https://pubmed.ncbi.nlm.nih.gov/33462485/
FinnGen consortium	Male infertility	994	https://www.finngen.fi/en
FinnGen consortium	Female infertility	9831	https://www.finngen.fi/en

### IVs selection

IVs associated with gut microbiota were carefully selected using rigorous screening criteria from multiple perspectives. Initially, we applied a relaxed significance threshold of *p* < 1 × 10^–5^ to identify SNPs that were potentially related to gut microbiota. Subsequently, we performed SNPs clumping by removing variants in linkage disequilibrium (LD, R^2^ > 0.001 and within 10,000 kb). This criterion has been widely applied in previous studies ^10, 11^. Moreover, the F-statistics of the chosen IVs exceeded the conventional threshold for weak instruments of F-statistic/10, indicating their strong predictive potential for gut microbiota^12^. Next, we extracted SNPs associated with gut microbiota from the outcome dataset, while discarding SNPs associated with the outcome (*p* < 1 × 10^–5^). We further standardized SNPs for exposure and outcome, eliminating palindromic effects and allelic inconsistent SNPs (e.g. A/G vs. A/C). To ensure the reliability of results, we performed MR analysis on gut microbiota with more than 2 SNPs^13^. Detailed information of the SNPs can be found in Table S1.

### Statistical analysis

The causal effect between gut microbiota and infertility was primarily assessed using the random-effect inverse variance weighted (IVW) method. As a main analytical approach, IVW estimates were considered to be in a relatively ideal state, with their effectiveness relying on the validity of all genetic variants and the ability to robustly detect causality^14, 15^. To acquire more reliable results across a wider range of scenarios, we employed other additional methods to further evaluate the exposure with significant estimates (IVW derived* p* < 0.05). These methods can provide more robust estimates under lenient conditions, although their efficiency may decrease. To address potential inconsistencies, a stricter instrument p-value threshold was applied^16^.

In addition, in order to fulfill key assumptions of our MR study design, specific sensitivity analyses were performed to evaluate and adjust for potential heterogeneity and pleiotropy, including the Cochran Q test, MR-Egger intercept, and MR-Pleiotropy Residual Sum and Outlier methods (MR-PRESSO). Cochran’s Q statistic was employed to detect heterogeneity, with a *p* value < 0.05, indicating heterogeneity of the results^17^. Furthermore, we calculated the MR-Egger intercept tests to appraise the influence level of directional pleiotropy resulting from invalid IVs on risk estimates^18^. A significantly deviating intercept value from the null (*p* < 0.05) suggested the presence of horizontal pleiotropy^19, 20^. Additionally, the presence of heterogeneous SNPs was examined using the MR-PRESSO global test. For further interpretation, a leave-one-out (LOO) analysis was performed to assess the influence of each exposure-associated SNP on the results, with the IVW analysis repeated iteratively by excluding a single SNP^19^.

## Results

### Mendelian randomization and sensitivity analysis between gut microbiota and male infertility

**Figure 2:**
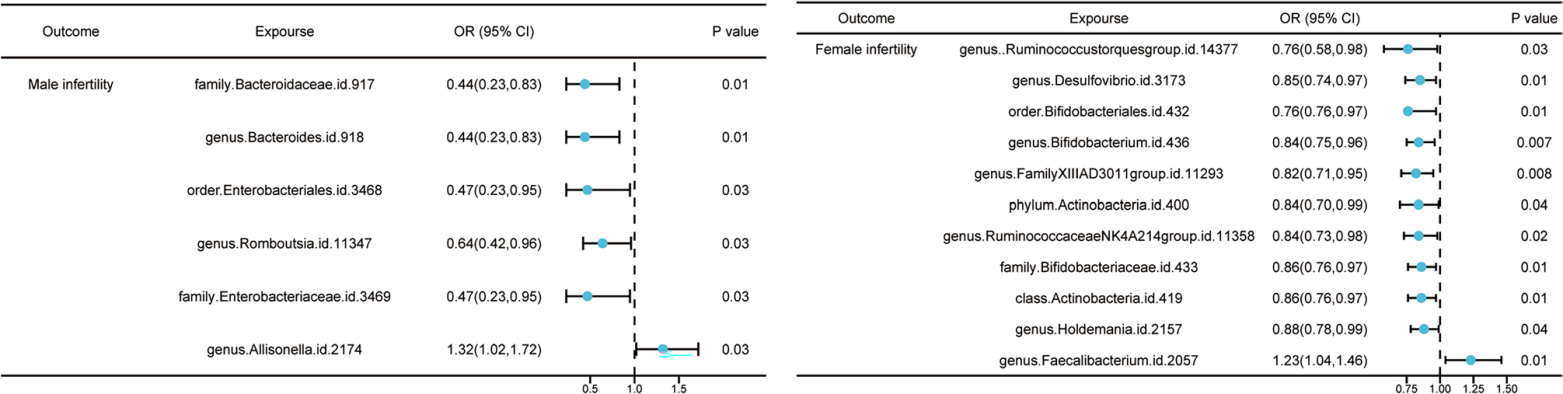
Causal effects from 196 gut microbiota on male/female infertility. The IVW approach was utilized for the summary of the MR estimates.

Among the gut microbiota analyzed in the MR study using microbiota-related SNPs, IVW initially identified 6 taxa that potentially have causal effects on male infertility (Tables S2, S3 and Fig. 2). Among these, 5 taxa showed a negatively association with male infertility, implying a potential protective role against male infertility, which were family.Bacteroidaceae.id.917 (odds ratio (OR) 0.44, 95% confidence interval (CI) 0.23–0.83, *p* = 0.01); genus.Bacteroides.id.918 (OR 0.44, 95% CI 0.23–0.83, *p* = 0.01); order.Enterobacteriales.id.3468 (OR 0.47, 95% CI 0.23–0.95, *p* = 0.04); genus.Romboutsia.id.11347 (OR 0.64, 95% CI 0.42–0.97, *p* = 0.03); family.Enterobacteriaceae.id.3469 (OR 0.47, 95% CI 0.23–0.95, *p* = 0.04). Notably, genus.Allisonella.id.2174 was positively associated with male infertility (OR 1.32, 95% CI 1.02–1.72, *p* = 0.04). Similar risk estimates were gained using the MR-Egger and weighted median approaches, although sometimes the associations were of no statistical significance. The consistent direction of the associations in MR-Egger and weighted median supported the robustness of the result. The P values from Cochran Q test and MR-Egger intercept MR-Egger intercept test were above 0.05, providing strong evidence for the absence of heterogeneity and pleiotropy (Tables S4, S5).

### Mendelian randomization and sensitivity analysis between gut microbiota and female infertility

For female infertility, IVW initially identified 11 taxa with potential causal effects on female infertility (Tables S6, S7 and Fig. 2). Apart from genus.Faecalibacterium.id.2057 causally increased the possibility of female infertility (OR 1.23, 95% CI 1.04–1.46, *p* = 0.02), the other 10 were also negatively associated with female infertility, indicating their protective role against female infertility, which include genus.Ruminococcustorquesgroup.id.14377 (OR 0.76, 95% CI 0.58–0.98, *p* = 0.03), genus.Desulfovibrio.id.3173 (OR 0.85, 95% CI 0.74–0.97, *p* = 0.02), order.Bifidobacteriales.id.432 (OR 0.86, 95% CI 0.76–0.97, *p* = 0.02), genus.Bifidobacterium.id.436 (OR 0.85, 95% CI 0.75–0.96, *p* = 0.008), genus.FamilyXIIIAD3011group.id.11293 (OR 0.82, 95% CI 0.71–0.95, *p* = 0.009), phylum.Actinobacteria.id.400 (OR 0.84, 95% CI 0.70–0.99, *p* = 0.04), genus.RuminococcaceaeNK4A214group.id.11358 (OR 0.85, 95% CI 0.73–0.98, *p* = 0.02), family.Bifidobacteriaceae.id.433 (OR 0.86, 95% CI 0.76–0.97, *p* = 0.02), class.Actinobacteria.id.419 (OR 0.86, 95% CI 0.76–0.97, *p* = 0.01), genus.Holdemania.id.2157 (OR 0.88, 95% CI 0.78–0.99, *p* = 0.04). In summary, the estimates derived from IVW are significant (*p* < 0.05) while the consistent direction of IVW, MR-Egger, and WM supporting the reliability of the result. With the exception of family.Bifidobacteriaceae.id.433 (*p* = 0.01) and phylum.Actinobacteria.id.400 (*p* = 0.003), all the *p* values for the MR-Egger intercept of the other 9 taxa were above 0.05, indicating the robustness of the results. Although horizontal pleiotropy was observed in IVW estimates, the significant causal associations were still validated using MR-Egger analysis (family.Bifidobacteriaceae.id.433 (*p* for MR-Egger = 0.002) and phylum.Actinobacteria.id.400 (*p* for MR-Egger = 0.001)). In addition, heterogeneity has been detected within the phylum level of the gut microbiota. Actinobacteria.id.400 (*p* for Cochran’s Q = 0.02) and class.Actinobacteria.id.419 (*p* for Cochran’s Q = 0.04) with female infertility (Tables S8, S9), the causal relations were still valid due to acceptable heterogeneity^21^. The MR-PRESSO global test showed no evidence of outlier pleiotropy or SNP outliers (*p* = 0.198 for female infertility; *p* = 0.741 for male infertility), further suggesting no proof of a pleiotropic effect was found.

## Discussion

In recent years, infertility has gained significant attention in research both domestically and internationally, as it poses a medical and social challenge that affects the well-being of populations. Despite its prevalence, the prevention of infertility remains a complex issue. Several studies have indicated a connection between infertility and gut microbiota^22^, but evidence regarding the causal role of gut microbiota in infertility is limited. In order to provide actionable strategies for prevention of infertility, we analyzed GWAS data comprehensively by means of a two-sample MR approach in this study. To the best of our knowledge, this is the first application of the most comprehensive gut microbiota GWAS data available to explore the causal relationship with infertility. Our analysis yielded conclusive findings regarding the causal associations between gut microbiota and infertility at various taxonomic levels, including phylum, class, order, family, and genus. Specifically, we identified 2 family taxa, 2 genus taxa and 1 order taxa (family.Bacteroidaceae.id.917, genus.Bacteroides.id.918, order.Enterobacteriales.id.3468, genus.Romboutsia.id.11347, family.Enterobacteriaceae.id.3469) that were linked to a low risk of male infertility, while1 genus taxa (genus.Allisonella.id.2174) was associated with a high risk of male infertility; Furthermore, we identified 6 genus taxa, 1 phylum taxa, 1 class taxa, 1 order taxa and 1 family taxa (genus.Ruminococcustorquesgroup.id.14377, genus.Desulfovibrio.id.3173, genus.Bifidobacterium.id.436, genus.FamilyXIIIAD3011group.id.11293, genus.RuminococcaceaeNK4A214group.id.11358, genus.Holdemania.id.2157, order.Bifidobacteriales.id.432, phylum.Actinobacteria.id.400, family.Bifidobacteriaceae.id.433 and class.Actinobacteria.id.419) that were linked to a low risk of female infertility, and 1 genus taxa (genus.Faecalibacterium.id.2057) was associated with a high risk of female infertility.

As the largest and most complex micro-ecosystem in the organism, the gut microbiota can exert influence on the digestive system and other organs, regulating various physiological functions including metabolism, cell development and immunity^23^. Meantime, advancements in high-throughput DNA sequencing technology have provided further insights into the composition and functions of gut microbes. Typically, the gut microbiota consists of a diverse combination of bacteria, which exist in a specific proportion. These bacteria interact with each other, exhibiting mutual restraint and interdependence, thereby establishing an ecological balance in terms of both quality and quantity.

However, an improper balance of related gut microbiota may be a crucial factor in the occurrence of infertility, which was in accordance with previous observational study conducted by Lundy et al.^24^. This study was the first comprehensive investigation of the male infertility microbiota, founding increased semen alpha-diversity, increased seminal aerococci, and decreased rectal anaerobic cocci in infertile men. Furthermore, Chinese scholars^25^ have identified that high-fat diet-induced imbalance in gut microbiota (A significant increase in Pseudomonas aeruginosa and Pseudomonas putida) led to decreased male fertility, independent of high-fat diet-induced metabolic disorders. They also observed a significantly higher proportion of Bacteroides and Prevotella in the fecal bacteria of infertile men compared to the controls^25^. Nevertheless, there is still a lack of strongly-proved RCTs to precisely determine the risks associated with gut microbiota disorder.

To date, there are no formal reports on the potential roles of these associated flora causing positive results in infertility. In the absence of evidence from RCTs and relevant observational studies, our study utilized extensive data from GWAS and genetic instruments to validate the causal effect of gut microbiota on infertility, eliminating confounding factors and concerns of reverse causality at the same time. The findings of our study offer significant support in the literature for future investigations in this area. Further research is necessary to confirm this causality and elucidate the underlying mechanisms. The subsequent section would focus on the mechanisms by which gut microbiota may increase the risk of infertility, aiming to guide future research.

### Gut microbiota and male infertility

We have observed that gut microbiota mentioned above has a broad impact on male infertility. In the following paragraph, we will discuss the mechanisms underlying gut microbiota dysbiosis.

Male infertility is often attributed to decreased sperm quality, and it is a well-known contributing factor. However, recent studies have indicated that gut microbiota dysbiosis can also play a significant role in male spermatogenesis and motility^25^. Firstly, a possible mechanism for this relationship is inflammatory response induced by endotoxin: (1) Endotoxin mediates the expression of pro-inflammatory factors: endotoxin, a cell wall lipopolysaccharide component found in Gram-negative bacteria (G-bacteria), plays a significant role in mediating the expression of pro-inflammatory factors. Dysfunctional intestinal flora, being a major source of circulating endotoxins, can lead to endotoxemia. This occurs when dead intestinal flora underwent autolysis, releasing endotoxin into the bloodstream. Toll-like receptor-4 (TLR-4) is expressed in human sperm and Sertoli cells, and endotoxin acts as a ligand for TLR-4, activating the TLR-4-myeloid differentiation primary response protein 88 (MyD88)-dependent pathway. Consequently, this activation leads to the release of various molecules, including interferon regulatory factor 3, mitogen-activated protein kinase, Jun N-terminal kinase, inducible nitric oxide synthase (iNOS), and type 1 interferon^26, 27^. These transcription factors collaborate to activate and regulate the expression of multiple pro-inflammatory factors. Notably, nuclear factor κB (NF-κB), interleukin (IL) 1β, IL-6, and tumour necrosis factor α (TNF-α) are among the activated inflammatory factors, which can cause vascular endothelial damage, disruption of the blood-testis barrier, and impair spermatogenesis and viability^28^,ultimately contributing to male infertility; (2) Endotoxin-mediated abnormal increase in iNOS: intestinal smooth muscle and intestinal capillary endothelial cells express iNOS, which is the rate-limiting enzyme for nitric oxide (NO) biosynthesis. Under the stimulation of endotoxin, pathogenic microorganisms can induce the binding of specific bacterial species or NF-κB to the promoter region of iNOS, thereby upregulating transcription of iNOS^28^. The abnormal increases in iNOS can disrupt the integrity of tight junctions within the blood-testis barrier, impair sperm-egg binding during fertilization, induce apoptosis in germ cells, and adversely affect sperm count. Moreover, elevated levels of NO inhibit sperm mitochondrial respiration, leading to mitochondrial hyperpolarization, the release of cytochrome C, and ultimately, the death of sperm cells^29, 30^. Besides, it also enhances the level of oxidative stress and promotes the production of chemically toxic substances. These substances, upon metabolic activation, can covalently bind to sperm DNA, resulting in the formation of adducts that damage DNA integrity, peroxidize sperm cell membrane lipids, and reduce sperm motility^31, 32^; (3) Endotoxin-mediated down-regulation the expression and function of β-defensins: β-defensins, synthesized by various epithelial cells, play a critical role as the host’s initial defense against infections on epithelial surfaces. Notably, the epididymis-specific defensin Bin1b is involved in sperm motility enhancement through its binding to the sperm head. Also, the presence of endotoxin can significantly reduce Bin1b expression and its affinity for sperm, thereby impairing sperm maturation and motility^33^. Additionally, abnormal expression of relevant genes due to dysbiosis of intestinal flora will further impair spermatogenic function. Disruptions in the Intestinal flora persistently inhibit the transcription of 17α-hydroxylase (P450c17), cholesterol side chain cleavage enzyme (P450scc), and steroid hormone synthesis acute regulatory protein genes necessary for testosterone synthesis, thereby suppressing 90% of testosterone production. Furthermore, dysregulation of the intestinal flora leads to significantly reduced expression of genes of the chromosome structure maintenance protein (SMC)family (encoding adhesion protein, condensin and SMC5/6 complex protein) and Sycp genes (encoding the association complex), which play key roles in sister chromatid association or segregation, chromosome condensation, repair of DNA double-strand breaks, and homologous chromosome linkage, and their decreased expression will result in reduced spermatogenesis. Dysbiosis-induced dysregulation of the intestinal flora also causes decreased expression of the Ggnbp2 gene, which is crucial for repairing meiotic DNA double-strand breaks in spermatocytes, consequently inhibiting spermatogenesis^34^. Moreover, reduced expression of the genes encoding core subunits of the respiratory chain on mitochondrial membranes, including MT-ND1, MT-ND2, MT-ND4, MT-ND5 and MT-ND4L, leads to increased sperm mitochondrial membrane potential and decreased sperm motility^35–37^. Furthermore, the diminished expression of the Crisp2 gene, essential for regulating Ca^2+^ influx during sperm capacitation and motility, has adverse effects on sperm motility^38^.

### Gut microbiota and female infertility

In female infertility, it has been observed that inflammation-related markers were elevated in female infertility patients, and this change was correlated with increased serum androgen levels. Meanwhile, there is a link between chronic inflammatory responses and alterations in gut microbiota^39^. In addition, previous studies have indicated that certain pathogenic bacteria in intestine, such as Pseudomonas, Streptococcus and Staphylococcus can produce lipopolysaccharide, which increases intestinal permeability. Consequently, lipopolysaccharide enters the bloodstream and triggers chronic systemic inflammation by activating the immune system. In infertile women, serum levels of lipopolysaccharide-binding protein were found to be positively correlated with C-reactive protein levels and IL-6 levels in follicular fluid, while being negatively correlated with progesterone production. This suggests that under conditions of high intestinal permeability, lipopolysaccharide can cause inflammation in the ovaries, leading to a reduction in progesterone production^39, 40^. Furthermore, research has confirmed that specific gut microbial communities have the ability to regulate the development of endotoxemia and inflammation, thereby contributing to the pathophysiological regulation of endotoxemia^41, 42^. Endotoxemia resulting from dysbiosis of the gut microbiota is also negatively associated with luteinizing hormone production, which can lead to luteinizing hormone deficiency^43^. Insufficient luteinizing hormone levels can disrupt the timely conversion of the endometrium and hinder the fertilization of the egg, resulting in infertility or habitual abortion.

The mechanisms described above suggest a potential causal association between gut microbiota and infertility. However, it is important to note that this assumption has not been thoroughly examined due to the lack of robust RCTs providing strong evidence. Currently, there is still a paucity of relevant RCTs investigating the specific relationship between gut microbiota and infertility, mainly due to the complex interactions between the host and gut microbiota. Further researches including RCTs are necessary to comprehensively understand the underlying mechanisms.

This MR analysis offers several notable advantages. Firstly, it represents the most comprehensive and systematic study conducted thus far to investigate the causal effects between gut microbiota and infertility, as it encompasses the analysis of 196 bacterial taxa. Secondly, rigorous MR analysis techniques were employed to address inherent limitations observed in previous studies, including concerns related to reverse causality and confounding interference. By utilizing multiple methods and conducting detailed analyses, the credibility and authenticity of our findings have been enhanced.

The current study inevitably possesses several limitations. Firstly, the SNPs that we selected as IVs may still be influenced by potential horizontal pleiotropy, as factors such as genetic inheritance, lifestyle, and environmental conditions can impact the gut microbiome, resulting in minor variations that may not be fully captured by the IVs. The current study is unable to determine the relevance of IVs to confounding. Secondly, the current study can’t comprehensively explore the entire spectrum of gut microbiota, from phylum to genus level, potentially missing other microbial species that could be causally linked to infertility, particularly those associated with increased risk. Thirdly, our data source primarily consists of GWAS summary statistics from individuals of Europe descent. Given the variations in the occurrence of specific traits across different racial and ethnic groups, driven by their diverse living environments and genetic backgrounds, caution should be exercised when generalizing the results to other populations with distinct lifestyles and cultural backgrounds. Therefore, future studies should enroll participants from diverse populations as possible to enhance the external validity of the findings. Fourthly, the accuracy of MR estimation is somewhat dependent on the sample size, so an enlarged sample size is also warranted to confirm the reliability of the results. MR investigations often require large sample sizes to increases statistical power and facilitate the discovery of additional genetic susceptibility loci^44^. At the same time, strong efforts should be taken to encourage the inclusion of the entire global ethnic population in genetic studies of this nature. Finally, Confounding factors such as diet and lifestyle can potentially influence the relationship between gut microbiota and infertility. We acknowledge this as a limitation of our research and recognize the need for further studies to better understand the impact of these factors on the relationship between gut microbiota and infertility. However, Mendelian randomization estimates based on genetic tools can also reflect certain estimated effects to some extent in our current study. Future research could employ more comprehensive approaches, such as long-term prospective cohort studies or interventional studies, to better assess the effects of diet and lifestyle on gut microbiota and infertility. Additionally, collaboration with other research fields, such as nutrition and lifestyle health, could provide further insights into addressing these confounding factors.

All in all, while the current study provides valuable insights, it is important to acknowledge these limitations and consider them when interpreting the results. Future research should address these limitations to further advance our understanding of the causal relationship between gut microbiota and infertility in a more comprehensive and diverse manner.

## Conclusions

In conclusion, we confirmed a causal link between gut microbiota and infertility through MR study utilizing publicly available GWAS summary data. Among these taxa, 5 were negatively associated with male infertility, which were family.Bacteroidaceae.id.917, genus.Bacteroides.id.918, order.Enterobacteriales.id.3468, genus.Romboutsia.id.11347, family.Enterobacteriaceae.id.3469. While 1 genus taxa (genus.Allisonella.id.2174) was positively associated with male infertility. In the case of female infertility, 6 genus taxa, 1 phylum taxa, 1 class taxa, 1 order taxa and 1 family taxa (genus.Ruminococcustorquesgroup.id.14377, genus.Desulfovibrio.id.3173, genus.Bifidobacterium.id.436, genus.FamilyXIIIAD3011group.id.11293, genus.RuminococcaceaeNK4A214group.id.11358, genus.Holdemania.id.2157, order.Bifidobacteriales.id.432, phylum.Actinobacteria.id.400, family.Bifidobacteriaceae.id.433 and class.Actinobacteria.id.419) were linked to a low risk of female infertility , while 1 genus taxa (genus.Faecalibacterium.id.2057) was associated with a high risk of female infertility. These findings of genetic prediction provide a valuable insight for early diagnosis, prevention, and treatment of infertility. It is important to note that while MR analysis puts up an outstanding performance at the exploration of etiology, further validation of these findings through strongly-proved RCTs is necessary.

## Supplementary Information

Supplementary Tables.

## Data Availability

After the article’s publication, all authors consent to sharing the study's data. The data will be obtained upon a reasonable request. All data supporting the results of this study are available from the corresponding authors upon request.

## Abbreviations

IVW: Inverse variance weighted
MR: Mendelian randomization
GWAS: Genome-wide association study
SNP: Single nucleotide polymorphism
RCT: Randomized controlled trials
LD: Linkage disequilibrium
WM: Weighted median
LOO: Leave-one-out
MR-PRESSO: MR-pleiotropy residual sum and outlier methods
TLR-4: Toll-like receptor-4
iNOS: Inducible nitric oxide synthase
SMC: Structure maintenance protein
